# 513. Evaluating a Novel Spray-on Dressing to Promote Healing in a Deep Partial Thickness Burn Model

**DOI:** 10.1093/jbcr/irag033.172

**Published:** 2026-04-06

**Authors:** Kai V Myree Lindsey, Abigail Bruhm, Lian Daniel N Manalo, William Sexter, Amanda Soo Ping Chow, Bonnie C Carney, Eriks Ziedins, Casey Meretta, Lauren T Moffatt, Jeffrey W Shupp

**Affiliations:** The Burn Center at MedStar Washington Hospital Center, Washington, DC; The Burn Center at MedStar Washington Hospital Center, Washington, DC; The Burn Center at MedStar Washington Hospital Center, Washington, DC; The Burn Center at MedStar Washington Hospital Center, Rockville, MD; The Burn Center at MedStar Washington Hospital Center, Washington, DC; The Burn Center at MedStar Washington Hospital Center, Washington, DC; Medstar Washington Hospital Center, Washington, DC; The Burn Center at MedStar Washington Hospital Center, Potomac, MD; The Burn Center at MedStar Washington Hospital Center, Washington, DC; MedStar Washington Hospital Center, Washington, DC

## Abstract

**Introduction:**

Burn wounds often require the use of a dressing to promote healing. Traditional dressings pose challenges stemming from their adhering properties and difficulty with removal. Some dressings create further damage to the dermis and epidermis causing trauma to tissues that are essential to healing and causing significant pain. It was hypothesized that the novel spray-on dressing, which was designed with ease of application and removal in mind, would be non-inferior in healing properties compared to existing dressings.

**Methods:**

Deep partial thickness burn injuries were created on a male Duroc pig. On day 0, the pig was shaved and depilated. Baseline biopsies, blood, and digital images were taken from the animal. Skin probe data was also collected to measure elasticity, stiffness, erythema, and melanin of uninjured skin. The animal was then subjected to a total of 6 burn wounds using a 9”x9” inch metal billet heated to 60°C. Post-injury, the same data were collected and the burns were dressed. Three wounds were dressed with antimicrobial foam dressing, serving as the control group. The remaining wounds were dressed with novel spray-on dressing, serving as the experimental group. Metrics described on day 0 were collected on days 3, 6, 9, and 13. Qualitative analysis of re-epithelialization was performed using digital images. Biopsies were embedded and stained. Epidermal thickness and rete ridge ratio were measured using ImageJ.

**Results:**

By day 10, most wounds were re-epithelialized. At day 13, the re-epithelization within the experimental group was comparable to the control group (97.7□2.4 vs. 92.1□6.9, *p*=.3). Elasticity, stiffness, and erythema measurements were not significantly different when compared to the uninjured skin or between groups (*p*>.05). The experimental wounds were significantly hypopigmented compared to the uninjured skin (*p*<.05), but not different from the control wounds. There was no significant difference in the epidermal thickness and rete ridge ratio between or within the groups over time (*p*>.05).

**Conclusions:**

The novel spray-on dressing was non-inferior in healing outcomes compared to a commonly used control dressing.

**Applicability of Research to Practice:**

The findings from this study show that a dressing with ease of application can function similarly to established dressings. The spray-on aspect of the dressing can serve as a solution for difficult-to-dress burns with complex geometry. Additional studies aimed at assessing the ease of application and pain mitigation during removal are underway.

**Funding for the study:**

This work was funded in part by Ionic Pharmaceuticals and the SBIR program (R44GM125412). This work was funded in part by award number KL2TR001432 from the National Center for Advancing Translational Science (NCATS/NIH).

